# Optimizing GPT-4 Turbo Diagnostic Accuracy in Neuroradiology through Prompt Engineering and Confidence Thresholds

**DOI:** 10.3390/diagnostics14141541

**Published:** 2024-07-17

**Authors:** Akihiko Wada, Toshiaki Akashi, George Shih, Akifumi Hagiwara, Mitsuo Nishizawa, Yayoi Hayakawa, Junko Kikuta, Keigo Shimoji, Katsuhiro Sano, Koji Kamagata, Atsushi Nakanishi, Shigeki Aoki

**Affiliations:** 1 Department of Radiology, Juntendo University Graduate School of Medicine, Tokyo 113-8421, Japan; 2 Clinical Radiology, Weill Cornell Medical College, New York, NY 10065, USA

**Keywords:** large language model (LLM), diagnostic imaging, neuroradiology, artificial intelligence in medicine, prompt engineering, confidence thresholds, GPT-4 Turbo, misdiagnosis reduction, AI diagnostic tools, clinical decision support

## Abstract

Background and Objectives: Integrating large language models (LLMs) such as GPT-4 Turbo into diagnostic imaging faces a significant challenge, with current misdiagnosis rates ranging from 30–50%. This study evaluates how prompt engineering and confidence thresholds can improve diagnostic accuracy in neuroradiology. Methods: We analyze 751 neuroradiology cases from the American Journal of Neuroradiology using GPT-4 Turbo with customized prompts to improve diagnostic precision. Results: Initially, GPT-4 Turbo achieved a baseline diagnostic accuracy of 55.1%. By reformatting responses to list five diagnostic candidates and applying a 90% confidence threshold, the highest precision of the diagnosis increased to 72.9%, with the candidate list providing the correct diagnosis at 85.9%, reducing the misdiagnosis rate to 14.1%. However, this threshold reduced the number of cases that responded. Conclusions: Strategic prompt engineering and high confidence thresholds significantly reduce misdiagnoses and improve the precision of the LLM diagnostic in neuroradiology. More research is needed to optimize these approaches for broader clinical implementation, balancing accuracy and utility.

## 1. Introduction

Large language models (LLMs) have demonstrated significant potential in processing textual information, often achieving performance levels comparable to human expertise. These models are increasingly being applied in medicine and offer valuable help in interpreting complex medical data. In general internal medicine, Hirosawa et al. reported a promising 93.3% accuracy for GPT-3 in diagnosing clinical vignettes [1]. Similarly, Chen et al. found that GPT-3 achieved 78.8% accuracy in symptom checks on a multilabel classification task using patient-reported symptoms [2]. In ophthalmology, Antaki et al. found that GPT-4 achieved 55.8% accuracy on a diagnosis task using fundus images and 42.7% on a treatment recommendation task [3]. In dermatology, Lallas et al. demonstrated a 51.5% sensitivity to diagnose early-stage skin cancer, and the inverse approach with classic pattern analysis improved to 83.6% [4]. For medical licensing exams testing broad clinical knowledge, Yang et al. reported promising results, with GPT-4 achieving 86.2% on the USMLE, 62.0% on AMBOSS, and 73.1% on the DRQCE exam [5].

In radiology, LLM has been reported to be indicated in various areas [6,7,8,9,10,11,12,13]. In terms of diagnostic accuracy performance, it is more modest. Bhayana et al. assessed ChatGPT performance on a board-style radiology examination, finding an overall precision of 69% [14]. Ueda et al. compared ChatGPT’s diagnostic performance from patient history and imaging findings, revealing a 61% diagnostic accuracy that supports its potential as a supplementary tool for clinicians [15]. Kottlors et al. explored the 68.8% feasibility of using ChatGPT for differential diagnosis based on imaging patterns [16]. Horiuchi et al. investigated the accuracy of ChatGPT-generated diagnoses from patient medical history and imaging findings in neuroradiology cases with a 50% lower accuracy than radiologists [17]. Suthar et al. conducted an in-depth evaluation of the accuracy of ChatGPT with the American Journal of Neuroradiology’s “Case of the Month”, reporting an overall diagnostic accuracy of 57.86% [18]. These reports suggest that LLM models achieve approximately 50–69% accuracy in identifying correct diagnoses from imaging findings. However, the 30–50% misdiagnosis rate is relatively high, which may be insufficient to assist healthcare professionals in making accurate clinical decisions. Physicians generally seek diagnostic tools that minimize the risks of misdiagnosis to ensure patient safety and effective treatment. The potential and limitations of LLM in medical applications highlight the need to improve accuracy and reduce misdiagnosis rates in clinical settings [19].

Prompt engineering, a method of giving precise instructions to LLMs, has shown effectiveness in eliciting desired responses and is reported to improve LLM performance in various applications [20]. This technique involves carefully crafting prompts that guide the LLM logically, ensuring that the model generates accurate and relevant outputs. Prompt engineering can help LLMs better understand complex queries and produce more useful responses by specifying the context, structure, and format of the response. In this study, we explore how prompt engineering can improve the diagnostic accuracy of LLM in medical imaging to reduce misdiagnosis rates.

In this study, we aim to utilize GPT-4 Turbo’s diagnostic abilities in medical imaging with prompt engineering techniques, particularly focusing on reducing misdiagnosis rates. By adopting these advanced technologies, we seek to improve the precision of diagnostic suggestions, using the capabilities of GPT-4 Turbo to address the challenges of accurately interpreting medical images.

## 2. Materials and Methods

This study was carried out using the checklist for artificial intelligence in medical imaging. It was exempted from institutional review board oversight because it used publicly available data.

### 2.1. Data Collection

Our methodology examined 751 publicly available neuroradiological cases from 2012 to 2023 from the American Journal of Neuroradiology (AJNR) Case of the Week Archives (https://www.ajnr.org/cow/by/diagnosis, accessed on 18 March 2024) [21]. The AJNR Case of the Week site separates clinical information, images, and diagnoses/explanations into separate tabs. GPT-4 Turbo accessed the indicated URL to retrieve clinical information and image findings as textual information without knowing the diagnosis name.

### 2.2. AI Model and Platform

We employed GPT-4 Turbo, an advanced version of the LLM developed by OpenAI [22]. The selection of GPT-4 Turbo for this study was based on its demonstrated superior performance in recent evaluations compared to other LLMs. According to a recent study, GPT-4 achieved a higher accuracy rate in medical diagnostics compared to other LLMs, validating its suitability for our research aimed at improving diagnostic precision [23]. Furthermore, GPT-4 has been found to generate more coherent and contextually relevant responses, which is crucial for accurate medical diagnosis and effective communication with clinicians. Based on these findings, we introduce GPT-4 Turbo, known for its enhanced capabilities and a vast 128 k context window, allowing comprehensive analysis within a single prompt.

To implement this, we employed the MD.ai Reporting/Chat application, which provides direct URL access to extract relevant clinical and imaging information, omitting diagnoses to test the diagnostic accuracy of GPT-4 Turbo [24]. MD.ai Chat is a reporting/chat application within the MD.ai platform that leverages LLMs such as GPT-3.5, GPT-4, and GPT-4 Turbo. One of the key features of MD.ai Chat is its support for natural language interactions, allowing healthcare professionals to engage in spoken dialogue with the model. This facilitates efficient and intuitive communication, making it easier to extract and understand complex clinical information. MD.ai Chat also provides direct URL access, allowing the retrieval of textual information necessary for clinical and imaging data analysis. Users can create customizable prompts for report interactions, tailoring the chat interface to their specific needs. This flexibility allows healthcare professionals to design the most relevant prompts for their clinical scenarios, enhancing the application’s utility.

### 2.3. Prompt Instruction

In this study, we employed three innovative prompt engineering strategies to improve the diagnostic precision of GPT-4 Turbo in neuroradiology. Drawing from cutting-edge engineering guides and research, we implemented role adoption, step-by-step thinking, and confidence level assessment [20,25]. Box 1 presents the detailed prompt used to guide the AI model in generating diagnostic suggestions.

#### 2.3.1. Role-Playing

This strategy involved programming GPT-4 Turbo to act as an expert in medical imaging diagnostics. Adopting the specialist role, the model shifted its processing from general data analysis to more focused, knowledge-based decision-making. This role-centered approach allowed LLM to prioritize critical and relevant information for disease diagnosis from various medical imaging modalities, such as CT, MRI, and X-rays. We hypothesized that by concentrating the focus of LLM on a specific domain, the quality and relevance of its diagnostic output would improve significantly, leading to higher precision in response and better alignment with clinical expectations.

#### 2.3.2. Step-by-Step Thinking

We directed GPT-4 Turbo to adopt a sequential diagnostic process that mirrors the decision-making steps that human experts typically take. The prompts guided the model in formulating an initial differential diagnosis based solely on clinical information, integrating imaging findings, and refining the initial diagnosis accordingly. This structured approach was designed to promote the gradual integration of data, improve the ability of the LLM to reason diagnostically, and increase the clarity and reliability of its diagnostic suggestions.

#### 2.3.3. Multiple Diagnostic Suggestions

Previous studies evaluating the accuracy of LLMs in medical imaging diagnostics typically required the LLM to provide only the most likely diagnosis [6,7,8,9,10,11,12,13]. However, in this study, we designed the prompts to instruct the LLM to provide the most likely diagnosis and four additional possible diagnostic suggestions, making a total of five potential diagnoses. This approach aims to increase the probability that the correct diagnosis is included among the suggestions, thereby reducing the likelihood of misdiagnosis. This strategy ensures that the LLM’s responses offer a broader spectrum of diagnostic possibilities, supporting the clinician’s decision-making process and enhancing the overall reliability of the AI system in a clinical setting.

#### 2.3.4. Confidence Assessment

The model was also instructed to perform self-monitoring by evaluating the confidence level of each diagnostic suggestion. This process involved the LLM evaluating its responses based on the difficulty of the task, the degree of consistency with the training data, and the consistency across its outputs. By quantitatively assessing confidence levels, we enabled a mechanism by which only high-confidence diagnostic suggestions were considered reliable. This method aimed to reduce misdiagnoses by ensuring that only the most probable and well-supported diagnoses were reported, improving the overall reliability of the diagnostic process.

These prompt engineering strategies were crucial to optimizing GPT-4 Turbo’s performance in diagnosing complex neuroradiology cases. The goal was to balance diagnostic accuracy with practical usability in a clinical setting.

Box 1 **Expert diagnostic prompt.**

# **Role**

You are an expert in medical imaging diagnosis with extensive experience interpreting various medical images, including CT, MRI, and X-rays. Your expertise includes identifying pathologies, understanding radiology clinical report contexts, and correlating to imaging findings with potential diagnoses proofread.

 

# **Request**

Along with the following Regulation prompt, present a refined list of five differential diagnoses, including the most probable diagnosis and four alternatives.

Each diagnosis should have a corresponding confidence level based on your comprehensive analysis.

  

# **Regulation**

Using the clinical information provided: {# URL of clinical information}, list five initial differential diagnoses.

Then, review the imaging findings: {# URL of image findings}, and update your diagnoses accordingly. Reflect on how the new data alters your assessment.

For each diagnosis in your updated list, assign a confidence level between 0% and 100%, considering the task's complexity and the extent to which clinical and imaging data support each diagnosis.

### 2.4. Evaluation of the Diagnostic Accuracy of GPT-4 Turbo

Two board-certified neuroradiologists with 15 and 28 years of clinical experience (T.A. and A.W) rigorously evaluated the diagnostic suggestions from GPT-4 Turbo. The evaluation involved a detailed review of GPT-4 Turbo’s response list, where each response was classified into one of three categories based on diagnostic accuracy: “Excellent”, “Good”, and “Insufficient”. This classification was performed post hoc by neuroradiologists who reviewed each response without prior knowledge of the AI confidence score or the difficulty level assessment provided by the model.

- Excellent: The top diagnostic suggestion matched the correct diagnosis exactly.
- Good: The correct diagnosis was among the top suggested candidates, indicating a useful but not precise match.
- Insufficient: The correct diagnosis was not listed among the suggested candidates, indicating a failure in the diagnostic process.

## 3. Results

### 3.1. Case Distribution Analysis

**Figure 1:**
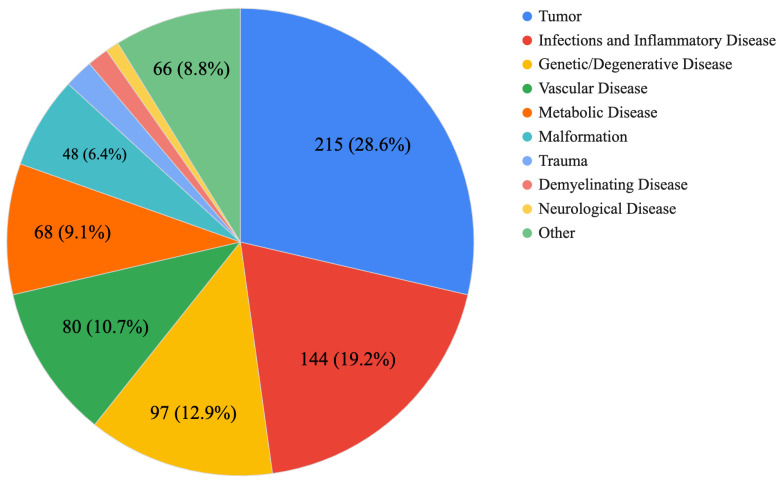
Proportion of total cases by disease category. This pie chart shows the distribution of 751 clinical cases in various disease categories.

Figure 1 classifies the 751 challenge cases into various disease categories. From this graph, it is recognized that there is a high frequency of challenges for tumors and demyelinating and inflammatory diseases, as well as genetic and degenerative diseases.

### 3.2. Diagnostic Performance Overview

**Figure 2:**
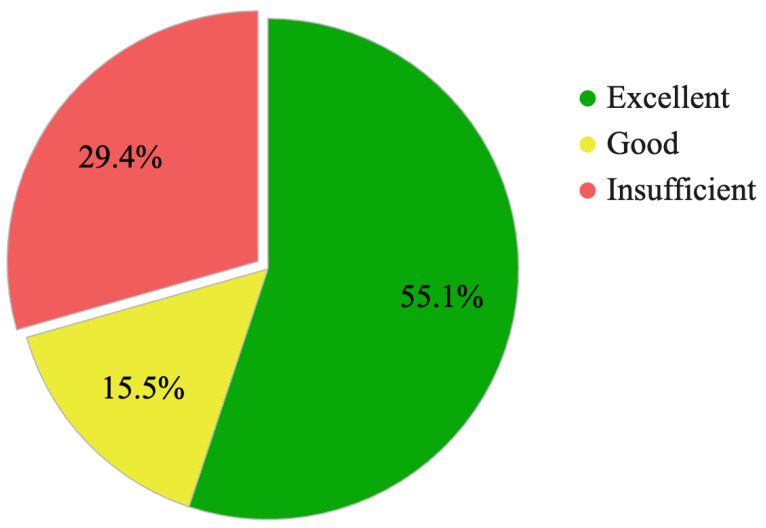
Evaluation of GPT-4 Turbo responses in 751 cases of neuroradiology.

Figure 2 and Table 1 illustrate the baseline diagnostic accuracy of GPT-4 Turbo in an analysis of 751 neuroradiological cases. The ‘Excellent’ rating, where the predicted top diagnosis precisely matched the ground truth, was obtained in 55.1% of cases. Furthermore, the ‘Good’ category, where the correct diagnosis was included among the top five predictions, accounted for 15.5% of cases. The total frequency of GPT-4 Turbo responses that included the correct diagnosis and helped clinical decision-making was 70.6%. However, the model produced ‘Insufficient’ responses in 29.4% of cases, where it failed to provide a useful candidate diagnosis.

The ‘Excellent’ category represents cases where the top diagnostic prediction matches the correct diagnosis. ‘Good’ indicates cases where the correct diagnosis was included within the top five diagnostic predictions. ‘Insufficient’ marks cases where the correct diagnosis was not included in the diagnostic predictions.

### 3.3. Impact of Confidence Thresholds on Diagnostic Accuracy

**Figure 3:**
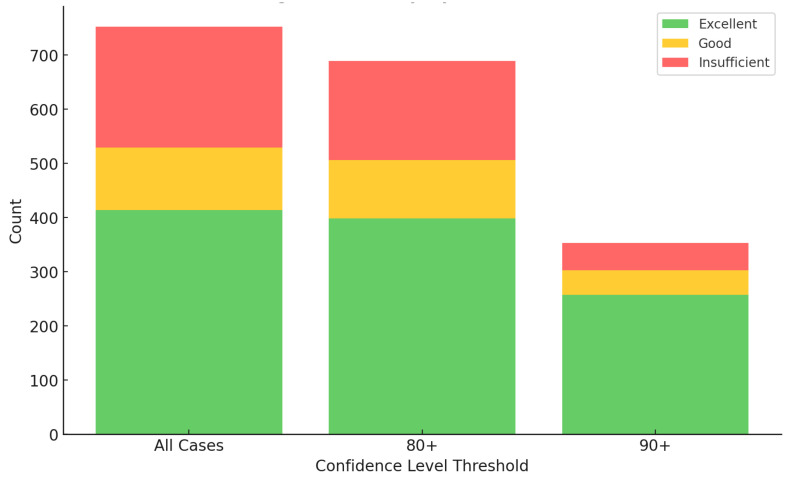
Impact of confidence thresholds on diagnostic performance.

Table 2 and Figure 3 show the effect of confidence threshold adjustments on diagnostic accuracy and acceptance rates. At the standard 60% confidence threshold, the ‘Excellent’ diagnoses rate remained steady at 55.1%, and the combined ‘Excellent + Good’ rate was 70.6%. In particular, the misdiagnosis rate was 29.4%. Increasing the confidence threshold to 90% markedly improved diagnostic precision: the misdiagnosis rate dropped to 14.1%, effectively halving the rate of diagnostic errors. During the same period, the rate of ‘excellent’ diagnoses increased to 72.9%. However, this rigorous threshold reduced the scope of the analysis to only 47% of the cases that met the high-confidence criteria.

Stacked bars represent the ‘Excellent’, ‘Good’, and ‘Insufficient’ diagnostic outcomes. This graph illustrates the diagnostic performance classified by confidence level threshold from 60 (all cases) to 90. At higher thresholds, the decay of ‘Excellent’ and ‘Good’ outcomes is slight. In contrast, ‘Insufficient’ decreases with increased thresholds, showcasing improved diagnostic reliability.

## 4. Discussion

The baseline precision of GPT-4 Turbo at 55.1% is in line with previous findings in this domain, underscoring the persistent challenge of improving diagnostic precision in complex medical fields. This study introduces innovative and prompt engineering strategies that reshape how LLMs process diagnostic data. Our prompt included three instructions: role-playing, step-by-step thinking, multiple diagnostic suggestions, and confidence assessment.

Role-playing is one of the fundamental techniques in prompt engineering. Tailored responses can be obtained by instructing the model to act as a specific entity, such as a historian or a scientist. For example, directing the model with “As a physician, evaluate the following treatment plan” can yield responses grounded in medical science.

Step-by-step prompt engineering is a crucial technique for optimizing AI performance. This approach, known as step-by-step thinking, involves guiding AI through a series of clear sequential steps to solve complex problems. By breaking down the problem-solving process, errors can be minimized at each stage, ultimately improving the accuracy of the final solution. Ye et al. demonstrated that prompt engineering is essential for enhancing the performance of LLMs [26]. They proposed using step-by-step reasoning templates to draw out complex inferential capabilities, thereby improving model performance.

Similarly, Sylvester and Reggia emphasized the importance of a step-by-step approach in high-level problem-solving with AI [27]. They developed a neural network framework that combines top-down and bottom-up approaches to achieve effective results, especially for tasks requiring ordered steps, such as card-matching problems. Scandura et al. highlighted the significance of step-by-step computational procedures in algorithm learning and problem-solving across various fields, showing how step-by-step methods contribute to effective problem resolution [28]. These studies underscore that step-by-step prompt engineering improves AI performance by facilitating structured and accurate problem-solving processes.

Providing multiple diagnostic suggestions instead of a single diagnosis is a technique that falls under adaptive prompting techniques. In the context of adaptive prompting, AI models are developed to adjust their responses based on the user’s input style and preferences. This personalization approach aims to make AI interactions more natural and user-friendly. For instance, if a user tends to ask concise questions, the AI adapts to provide concise answers, and vice versa. This development is particularly promising for enhancing user experience in AI-driven applications such as virtual assistants and chatbots. Applying this concept to a medical diagnosis and providing multiple potential answers can be considered an adaptive prompting technique. By offering several diagnostic suggestions, the AI adapts to the clinician’s needs, who may need various options for the best possible diagnosis. This method supports the clinician’s decision-making process and improves the overall accuracy and reliability of the AI system in a clinical setting. By expanding from a single diagnostic prediction to a set of five, the probability of including the correct diagnosis increased to 70%, and the instances of insufficient responses were reduced to 30%. In medical diagnosis, where LLMs assist doctors, the risk of misdiagnosis can be reduced if LLMs provide five potential diagnoses rather than just one. Offering multiple diagnostic suggestions increases the probability of including the correct diagnosis, thus reducing the chance of misdiagnosis. Zheng et al. found that the accuracy of LLM responses greatly depends on the design of the prompts [29]. Using methods like Progressive-Hint Prompting, LLMs are guided through a series of steps, helping them to get closer to the correct answer. This approach involves generating multiple potential answers and using these as hints to refine subsequent responses, ultimately improving the final accuracy. Huang et al. showed that LLMs can improve their reasoning skills by using high-confidence answers that they generate themselves. By producing multiple answers and selecting the most reliable ones, LLMs improve their accuracy and reliability [30]. Savage et al. demonstrated that providing multiple diagnostic suggestions helps physicians better assess and understand LLM diagnoses. This approach makes it easier for physicians to review the LLM diagnostic process, improving diagnostic accuracy and gaining greater trust in the LLM [31].

Implementing systems that consider confidence thresholds is a key aspect of adaptive modeling techniques. Confidence thresholds involve evaluating the model’s confidence in its predictions and using this information to make decisions about resource allocation and response handling. By focusing on predictions with high confidence and addressing those with low confidence differently, models can enhance their accuracy and efficiency. Research highlights the application of confidence thresholds in various contexts, such as uncertainty-aware in-context learning, which filters out responses with high uncertainty to improve reliability [32]. Generating confidence scores based on agreement between different prompts creates more accurate predictions, which is essential for decision-making processes [33]. Self-improvement techniques, where models use high-confidence answers for further learning, enhance reasoning abilities without needing labeled data [30]. Additionally, Pareto’s optimal self-supervision aids in calibrating model responses by providing risk scores for each answer, boosting accuracy in critical fields such as biomedicine [34]. By integrating confidence thresholds into adaptive modeling techniques, LLMs deliver more accurate and reliable results, making them more effective for various applications [35].

In our study, a novel approach was introduced that uses confidence thresholds to filter LLM responses. This added an analytical layer that effectively halved the frequency of incorrect diagnoses from 29.4% to 14.1%, highlighting the potential of this approach to improve the clinical utility of LLMs. Implementing these thresholds led to a substantial decrease in insufficient responses, from 221 to 50, in a dataset of 751 cases. However, this method required greater human intervention in 53% of cases where the LLM did not meet the high-confidence criteria, revealing a significant trade-off between accuracy and practical usability. The model automatically recognizes uncertainty in scenarios where responses are not obtained and filters out high-uncertainty responses, enhancing reliability.

The success of diagnostic efforts depends on human expertise and the quality of data supplied to the LLM. High confidence thresholds significantly reduce the risk of incorrect diagnoses and restrict the number of cases the LLM can effectively address. Requiring medical professionals to evaluate multiple diagnostic proposals introduces additional cognitive load, affecting clinical workflows and decision-making processes. It is important to note that pursuing excessively high confidence thresholds does not yield beneficial results. Although setting the confidence threshold at 100% ensures perfect accuracy, it covers only 8 out of 751 cases, representing a mere 1% reduction in radiologists’ workload. This outcome demonstrates that current AI technology cannot replace the capabilities of radiologists.

This study focused on precision in scenarios where sufficient information was provided for diagnosis, indicating the need for further research on LLM performance with real-world data, which may be incomplete or atypical, and in domains beyond neuroradiology. Future research should enhance LLM accuracy by expanding learning data, especially for tasks with low confidence levels, and implementing few-shot learning as part of prompt engineering to refine LLM reasoning processes by providing examples of human thought processes. Optimizing these methods to increase the proportion of high-confidence cases is crucial for future research.

To address the concerns raised about the composition of the AJNR Case of the Week Archive, we acknowledge that the cases included in our study are more specialized compared to those typically encountered in routine clinical practice. The AJNR Case of the Week Archive is curated by editors who select cases that are educational and designed to improve diagnostic skills by presenting unique and challenging scenarios. Consequently, the frequency and types of disease in the archive do not represent the actual distribution seen in everyday clinical settings. This selection bias is a limitation of our study, and we have taken it into account in our analysis. For example, when comparing the cases targeted in this study with those encountered in routine clinical practice, particularly in the context of trauma, there are several notable differences. The number of trauma cases in our dataset is relatively low. Additionally, the ability to detect abnormalities, which is crucial in trauma imaging, was not evaluated in this study. These factors should be considered limitations.

## 5. Conclusions

Prompt engineering strategies, including the appropriate setting of confidence thresholds, improve its diagnostic assistance capabilities. Currently, the simple diagnostic accuracy of GPT-4 Turbo in neuroradiology is not ideal, but AI-assisted image diagnosis and clinical decision-making with increased reliability are a promising path to improving medical outcomes. Our research results will encourage the continued development and refinement of these strategies to maximize the potential of AI in improving healthcare outcomes.

## Figures and Tables

**Table 1:** Breakdown of GPT-4 Turbo tasks in each disease category in neuroradiological cases.

Disease Category	GPT4-Turbo Response Results			Proportion of Total Cases
	Excellent	Good	Insufficient	
Tumor	0.442	0.191	0.367	0.29 (215)
Demyelinating Disease	0.727	0.000	0.273	0.01 (11)
Infections and Inflammatory Disease	0.535	0.153	0.313	0.19 (144)
Vascular Disease	0.688	0.150	0.163	0.11 (80)
Genetic/Degenerative Disease	0.515	0.134	0.351	0.13 (97)
Trauma	0.667	0.267	0.067	0.02 (15)
Metabolic Disease	0.735	0.088	0.176	0.09 (68)
Malformation	0.563	0.229	0.208	0.06 (48)
Neurological Disease	0.571	0.000	0.429	0.01 (7)
Other	0.576	0.106	0.318	0.09 (66)
Total	0.551	0.154	0.294	1.00 (751)

**Table 2:** Diagnostic accuracy of GPT-4 Turbo at Varying Confidence Thresholds.

Confidence Threshold	Excellent (%)	Good (%)	Insufficient (%)	Adoption Rate
≥60%	55.1	15.5	29.4	100% (751/751)
≥70%	55.3	15.5	29.2	99% (746/751)
≥80%	57.9	15.8	26.3	92% (689/751)
≥90%	72.9	13.0	14.1	47% (354/751)
1	87.5	12.5	0.0	1% (8/751)

## Data Availability

The logs of conversations with GPT-4 Turbo from this study will be made publicly available on GitHub at https://github.com/aki-wada/GPT4-Turbo, accessed on 17 July 2024.
